# Reversal of Cushing syndrome by antibody-mediated neutralization of ACBP/DBI

**DOI:** 10.15698/cst2026.01.314

**Published:** 2026-01-26

**Authors:** Zhe Shen, Hui Pan, Xiaolian Deng, Oliver Kepp, Isabelle Martins, Guido Kroemer

**Affiliations:** 1 Université Paris Cité, Centre de Recherche des Cordeliers, Equipe labellisée par la Ligue contre le cancer, Inserm U1138, Sorbonne Université, Paris, France; 2 Université Paris Saclay, Metabolomics and Cell Biology Platforms, UMS AMMICa, Gustave Roussy Institut, Villejuif, France; 3 Faculté de Médecine, Université Paris-Saclay, Le Kremlin Bicêtre, Paris, France; 4 Department of Hematology/Institute of Hematology, West China Hospital, Sichuan University, Chengdu, China; 5 Institut du Cancer Paris CARPEM, Department of Biology, Hôpital Européen Georges Pompidou, AP-HP, Paris, France

**Keywords:** endozepin, hypercortisolism, neuroendocrine, obesity

## Abstract

Cushing syndrome (CS) is caused by an increase in endogenous or exogenous glucocorticoids, leading to major alterations in body composition, including visceral obesity, sarcopenia, osteoporosis, type 2 diabetes, and dyslipidemia. Cardiovascular complications resulting from CS are often lethal. We previously demonstrated that CS induced by oral corticosterone (CORT) supplementation in mice can be prevented by inhibition of the peptide hormone acyl-CoA binding protein (ACBP), encoded by the gene diazepam binding inhibitor (DBI). Here, we investigated whether ACBP/DBI inhibition could be used to treat, rather than prevent, CS. To this end, we initiated treatment with anti-ACBP/DBI monoclonal antibodies (mAbs) in mice three weeks after the start of CORT supplementation, when hyperphagia and body weight gain were already established. Two anti-ACBP/DBI mAbs, 7G4a (specific for mouse ACBP/DBI only) and 82 (which recognizes both mouse and human ACBP/DBI), were able to normalize food intake and halt weight gain in mice under continuous CORT treatment. In addition, both mAbs attenuated CORT-induced sarcopenia, adiposity in inguinal, perigonadal, and visceral fat depots, and fully restored metabolic parameters, including type-2 diabetes, insulinemia, free fatty acids, triglycerides, and liver transaminases. In conclusion, neutralization of ACBP/DBI may serve as an effective therapeutic strategy for the treatment of established CS.

## INTRODUCTION

Glucocorticoids (GCs) exert profound effects on whole-body metabolism and physiology, orchestrating complex adaptive and maladaptive responses that contribute to the pathogenesis of Cushing syndrome (CS) 1–3. Chronic GC excess from internal sources (such as adrenocortical carcinomas or pituitary adenomas inducing glucocorticoid production by the normal adrenal cortex due to the action of the hormone corticotropin, best known as ACTH) or external (iatrogenic) administration, leads to hallmark features of CS including hyperphagia, adipose tissue redistribution (lipodystrophy), muscle atrophy (sarcopenia), insulin resistance, dyslipidemia, depression, cognitive decline, and immunosuppression 4, 5.

**Figure 1:**
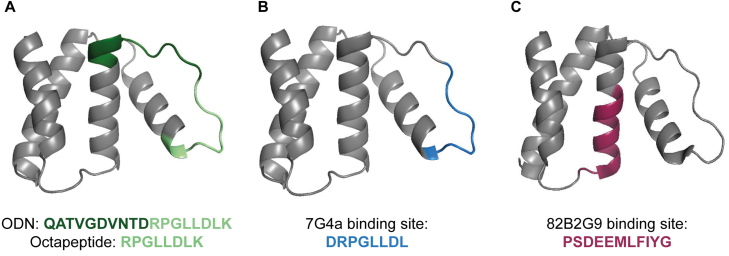
Comparison of domains and epitopes in ACBP/DBI. **(A)** Location of the octadecaneuropeptide (ODN, green) which includes the octapeptide (light green) within mouse ACBP/DBI protein. **(B)** Epitope recognized by the tool monoclonal antibody 7G4a (blue), which is specific for mouse ACBP/DBI. **(C)** Epitope recognized by monoclonal antibody 82B2G9 (red), which cross-reacts with both mouse and human ACBP/DBI.

Despite extensive characterization of GC receptor (GR)-mediated transcriptional networks, the identification of critical downstream soluble mediators that propagate GC-induced systemic metabolic alterations remains incomplete. Recent evidence implicates acyl-CoA binding protein/diazepam binding inhibitor (ACBP/DBI), an endogenous peptide ligand of the gamma-amino butyrate acid (GABA) Type A (GABA ${}_{A}$ ) receptor 6, 7, as a pivotal effector linking GC signaling to metabolic and body composition changes 8. Constitutive genetic deletion or pharmacological neutralization of ACBP/DBI has been shown to prevent the development of key features of CS in mouse models 9, 10, yet whether targeting ACBP/DBI after the onset of CS can reverse established metabolic dysfunction has not been elucidated.

This study investigates the therapeutic potential of ACBP/DBI neutralization in a well-established murine model of CS induced by oral administration of corticosterone (CORT), which is the most abundant endogenous glucocorticoid in rodents 11. Using bi-weekly administration of monoclonal antibodies (mAbs) specific for ACBP/DBI following the onset of GC excess, we demonstrate robust normalization of hyperphagia, arrest of weight gain, and reversal of pathological body composition. Furthermore, ACBP/DBI blockade markedly ameliorates GC-induced metabolic derangements such as hyperinsulinemia, insulin resistance, dyslipidemia, and hepatic injury markers. These findings build upon previous prophylactic data, positioning ACBP/DBI neutralization as a viable therapeutic strategy for established CS.

## RESULTS

### ACBP/DBI neutralization reverses CORT-triggered hyperphagia and halts CORT-induced weight gain

**Figure 2:**
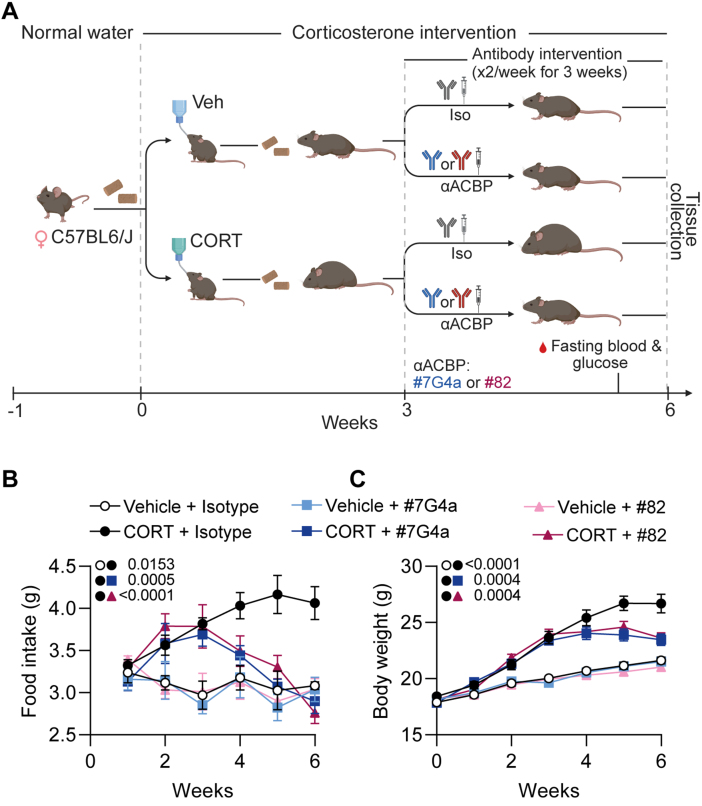
Neutralization of ACBP/DBI attenuates corticosterone-induced hyperphagia and weight gain in mice. **(A)** Experimental design for the pharmacological neutralization of ACBP/DBI in corticosterone (CORT)-treated mice. Female C57BL/6J mice were administered CORT (100 μ gml ${}^{-1}$ in drinking water) or vehicle (Veh) for 3 weeks. Beginning at week 3, mice received intraperitoneal injections twice a week of either an isotype control antibody (5 mg kg ${}^{-1}$ body weight) or one of two neutralizing anti-ACBP/DBI monoclonal antibodies ( α ACBP: #7G4a or #82, each administered at 5 mg kg ${}^{-1}$ body weight) for 3 additional weeks. Blood glucose was measured after fasting, and tissues were harvested at week 6. Created with BioRender.com. **(B)** Longitudinal measurement of daily food intake per mouse (g/day) was monitored in the indicated groups (n=8/group). **(C)** Body weight was monitored weekly (n=8/group). All data represent the mean ± SEM. Statistical comparisons were performed using the TumGrowth application (https://kroemerlab.shinyapps.io/TumGrowth/).

C57BL/6J mice received either the CS-inducing intervention (CORT diluted in ethanol added to the drinking water *p.o.*) or the vehicle control (an equivalent quantity of ethanol) at the age of 12 weeks for 6 weeks. Three weeks after initiation of CORT or vehicle treatment, mice received twice-weekly intraperitoneal (i.p.) injections of anti-ACBP/DBI-specific mAbs (either the IgG2A mAb 7G4a or the IgG1 mAb 82) 12, 13, which recognize distinct, non-overlapping epitopes in the ACBP/DBI protein (Figure 1). As a control, mice received vehicle (PBS) with their respective IgG isotype control mAbs instead of mAb 7G4a or 82B2G9 (abbreviated as “82”), as indicated in the schematic overview of the study (Figure 2**A**). Importantly, both food intake and body weight were significantly increased after 3 weeks of CORT treatment, prior to mAb administration (Figure 2**B, C**). Subsequently, injection of isotype control mAbs failed to mitigate further increases in food intake and weight. In contrast, both anti-ACBP/DBI-specific mAbs consistently normalized food intake during the three-week treatment period (Figure 2**B**) and arrested weight gain (Figure 2**C**), indicating that ACBP/DBI neutralization exerts therapeutic effects in established Cushing syndrome.

### ACBP/DBI neutralization attenuates CORT-induced changes in body composition

**Figure 3:**
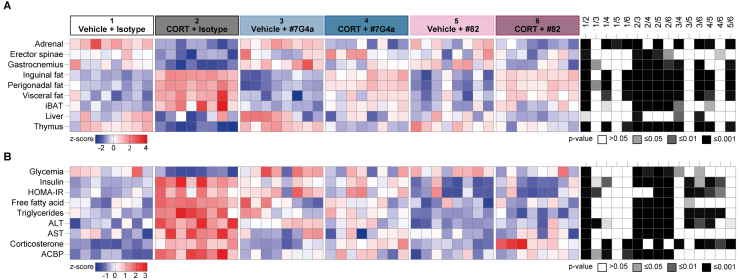
Neutralization of ACBP mitigates corticosterone-induced metabolic alterations. Female C57BL/6J mice were treated with vehicle or corticosterone (CORT, 100 μ gml ${}^{-1}$ in drinking water) for 3 weeks. Beginning at week 3, mice received intraperitoneal injections twice a week of either an isotype control antibody (5 mg kg ${}^{-1}$ body weight) or one of two neutralizing anti-ACBP/DBI monoclonal antibodies ( α ACBP: #7G4a or #82, each administered at 5 mg kg ${}^{-1}$ body weight) for 3 additional weeks. **(A)** A heatmap shows the standardized deviations (z-scores) of individual tissue weights normalized to body weight, including adrenal glands, erector spinae, gastrocnemius, inguinal white adipose tissue (iWAT), perigonadal fat, visceral fat, interscapular brown adipose tissue (iBAT), liver, and thymus. **(B)** A second heatmap presents the z-scores of circulating metabolic and hormonal biomarkers, including glycemia, insulin, HOMA-IR, free fatty acids, triglycerides, alanine aminotransferase (ALT), aspartate aminotransferase (AST), corticosterone, and plasma ACBP. Statistical comparisons were performed using pairwise two-tailed Wilcoxon tests with false discovery rate (FDR) correction for multiple comparisons. All measurements were conducted at the end of the 6-week protocol. P values are indicated in the statistical heatmaps on the right.

At endpoint, all mice were euthanized and specific organs were weighed to calculate their relative contribution to body mass. As expected, CORT significantly reduced the relative mass of the adrenal glands and representative skeletal muscles (Musculus erector spinae and Musculus gastrocnemius), while increasing the relative mass of white adipose tissue (WAT) in the inguinal, perigonadal, and visceral areas, as well as that of interscapular brown adipose tissue (iBAT) (Figure 3).

**Figure 4:**
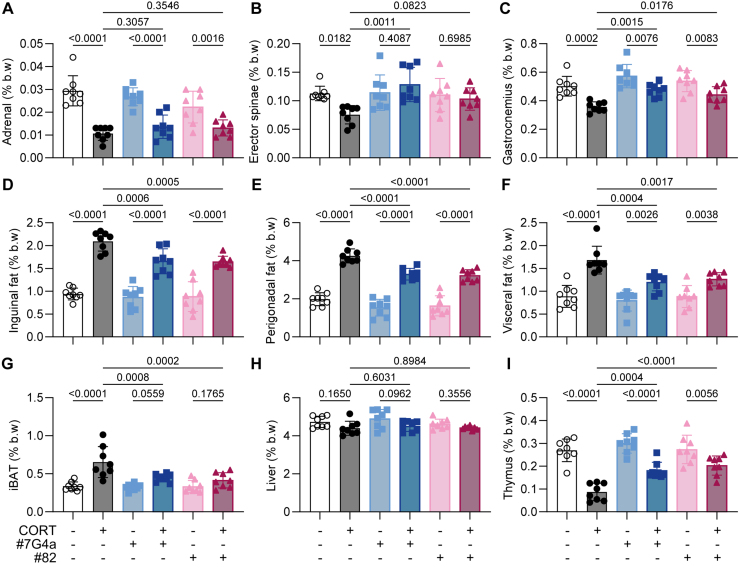
Neutralization of ACBP/DBI counteracts CORT-driven tissue atrophy and adipose expansion. Mice were processed as described in Figure 2, and tissue weights were measured at the end of the 6-week protocol. **(A–I)** The weight of adrenal glands **(A)**, erector spinae **(B)**, gastrocnemius muscle **(C)**, inguinal white adipose tissue (iWAT, **D**), perigonadal fat **(E)**, visceral fat **(F)**, interscapular brown adipose tissue (iBAT, **G**), liver **(H)**, and thymus **(I)** was quantified and normalized to total body weight. Statistical comparisons were performed using one-way ANOVA followed by multiple comparisons corrected using the original FDR method. All bar graphs represent mean ± SEM. P values are indicated above the bars. Significance thresholds: p<0.05, p<0.01, p<0.001, p<0.0001.

Both mAbs neutralizing ACBP/DBI failed to prevent adrenal gland involution (Figure 4), consistent with the CORT-induced blockade of ACTH secretion 14. However, ACBP/DBI neutralization significantly mitigated CORT-induced sarcopenia and thymus atrophy. Moreover, ACBP/DBI blockade attenuated the expansion of iBAT and WAT in all locations, indicating a net mitigation of the CORT-induced alterations in body composition (Figure 4).

### ACBP/DBI neutralization normalizes CORT-induced hyperinsulinemia, dyslipidemia and hepatic transaminases

At endpoint, we also investigated the effects of ACBP/DBI neutralization on several plasma parameters, including CORT-induced hypoglycemia, hyperinsulinemia, HOMA-IR (Homeostatic Model Assessment for Insulin Resistance), as well as proxies of dyslipidemia (increased free fatty acid and triglyceride concentrations) or liver damage (elevated alanine transaminase [ALT] and aspartate transaminase [AST]) (Figure 3).

**Figure 5:**
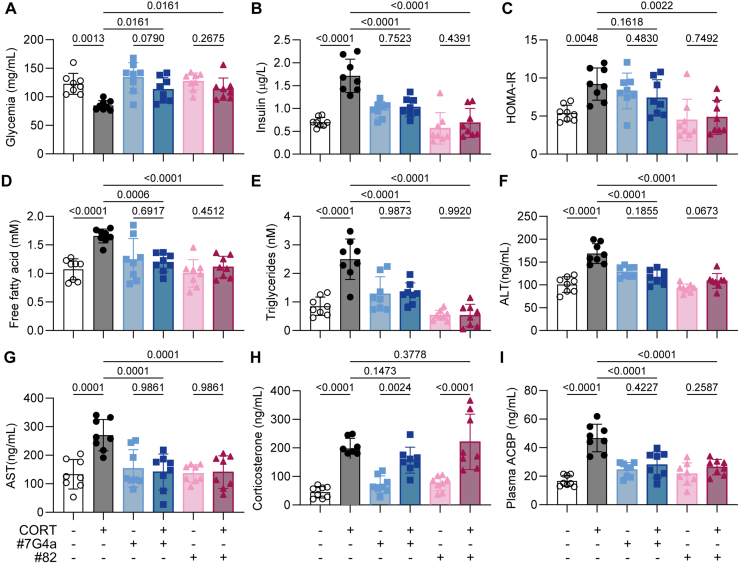
Neutralization of ACBP/DBI mitigates CORT-induced metabolic dysfunction and liver injury. Mice were processed as described in Figure 2, and plasma samples were collected at the end of the 6-week protocol. **(A–C)** Plasma glycemia **(A)**, insulin **(B)**, and HOMA-IR index **(C)** were measured to assess glucose metabolism. **(D–G)** Plasma levels of free fatty acids **(D)**, triglycerides **(E)**, alanine aminotransferase (ALT, **F**), and aspartate aminotransferase (AST, **G**) were quantified as markers of lipid metabolism and liver function. **(H–I)** Corticosterone **(H)** and plasma ACBP **(I)** concentrations were measured as indicators of endocrine regulation. Statistical comparisons were performed using one-way ANOVA followed by multiple comparisons corrected using the original FDR method. All bar graphs represent mean ± SEM. P values are indicated above the bars. Significance thresholds: p<0.05, p<0.01, p<0.001, p<0.0001.

As expected 9, neither mAb 7G4a nor mAb 82 affected plasma CORT concentrations, but both significantly reduced ELISA-detectable levels of circulating ACBP/DBI. In addition, ACBP/DBI neutralization fully normalized glycemia, hyperinsulinemia, insulin resistance, dyslipidaemia, and circulating transaminases (Figure 5). These results support the idea that CS-associated metabolic syndrome can be fully reversed by ACBP/DBI inhibition.

## DISCUSSION

Our previous work revealed that constitutive inactivation of the ACBP/DBI system, either through whole-body or hepatocyte-specific Dbi knockout, or by mutation of the ACBP/DBI receptor (*i.e.*, the F77I mutation in the γ 2 subunit of the GABA A-type receptor), fully prevented the metabolic signs of CS induced by CORT treatment 9. In addition, pretreatment of female or male mice with anti-ACBP/DBI mAb before CORT supplementation was able to prevent most, if not all, the manifestations of CS 9. Here, we extend these findings by showing that anti-ACBP/DBI mAbs administered after CS induction exert not only prophylactic but also therapeutic effects. Given that our previous work demonstrated comparable protective effects of ACBP/DBI neutralization in both male and female mice 9, the use of females in the present study does not introduce a sex-related bias.

Given these results, it appears surprising that major metabolic effects of glucocorticoids would rely on the downstream action of ACBP/DBI. This raises the question of whether other glucocorticoid-induced factors might play similar roles as ACBP/DBI. Previous reports have involved a variety of tissue hormones as secondary mediators of glucocorticoids. Thus, glucocorticoids upregulate myostatin in muscle cells, and myostatin deficient mice fail to manifest muscle atrophy in response to glucocorticoids 15–17. Receptor activator of NF- κ B ligand (RANKL), which is produced by osteocytes, is strictly required for osteoporosis and osteopenia induced by glucocorticoids 18. However, RANKL expression is not directly modulated by glucocorticoids 18, suggesting that it plays a permissive rather than an active role in the bone effects of glucocorticoids. In sharp contrast, interleukin-10 and annexin A1 are actively induced by glucocorticoids in various cell types to mitigate immune responses and inflammation, respectively 19–21. However, interleukin-10 administration prevents insulin resistance in mice 22, while its knockout exacerbates diabetes-associated bone loss 23. Moreover, suppression of annexin A1 expression in mice causes enhanced adiposity and diabetes 24. These findings suggest that neither annexin A1 nor interleukin-10 mediate the obesogenic, diabetogenic action or bone weakening effects of glucocorticoids. In the present work, we did not directly quantify glucocorticoid receptor (GR) or mineralocorticoid receptor (MR) expression in adipose tissue or liver, and we therefore cannot exclude a contribution of local receptor regulation. However, the absence of changes in circulating corticosterone concentrations upon ACBP/DBI neutralization suggests that its protective effects are predominantly exerted downstream of glucocorticoid receptor activation rather than via modulation of GR/MR abundance, a hypothesis that will require dedicated mechanistic studies.

Long-term exposure to glucocorticoids, whether due to endogenous, apparently ’subclinical’ hypercortisolism (also referred to as ’minimal autonomous cortisol secretion’) or prolonged pharmacological treatment, can induce several hallmarks of aging, even in the absence of overt Cushing syndrome 25, 26. ‘Subclinical’ hypercortisolism is associated with difficult-to-manage diabetes and hypertension, as well as age-related conditions such as sarcopenia, osteoporosis, chronic kidney disease, cardiovascular disease, and increased susceptibility to infections 27–30. In this context, it is noteworthy that levels of ACBP/DBI, increase with age in humans 31, 32, particularly preceding or during the onset of age-related diseases 7, 33–35. In murine models, neutralization of ACBP/DBI mitigates or prevents a broad spectrum of age-associated pathologies, including metabolic syndrome 12, steatohepatitis 36, kidney dysfunction 32, myocardial infarction 36, heart failure 32, 37, cancer 33, 35, 38, 39, and osteoarthritis 13. These findings raise the intriguing possibility that ACBP/DBI may mediate at least some of the pro-aging effects associated with chronic glucocorticoid exposure in humans.

In summary, ACBP/DBI currently emerges as a key downstream mediator of glucocorticoid action, particularly with respect to systemic metabolism and body composition. Furthermore, ACBP/DBI may contribute to the hypothesized pro-aging effects of prolonged glucocorticoid exposure. Moving forward, it will be crucial to investigate the metabolic consequences of ACBP/DBI activity downstream of its modulation of chloride and bicarbonate fluxes via the GABA ${}_{A}$ receptor, in order to fully elucidate its broad physiological roles.

## MATERIALS AND METHODS

### Mouse experiments

C57BL/6J WT mice were housed under temperature-controlled conditions and provided with food and water *ad libitum*. All experimental procedures complied with FELASA guidelines and received approval from the local ethics committee (protocol no. 2024_040_50288). Mice received intraperitoneal injections twice a week of anti-ACBP/DBI monoclonal antibodies (mAb 7G4a or mAb 82) at a dose of 5 mg/kg body weight for three weeks, and control animals were injected with an isotype-matched antibody at the same concentration.

### Glycemia and HOMA-IR

Blood samples were obtained via tail incision, and glycemia was assessed using a calibrated glucometer (Accu-Chek Performa). Mice were monitored throughout the experiments, and hypoglycemic episodes were prevented by administering a 20% glucose solution when necessary. The HOMA-IR index was calculated using the standard formula: fasting blood glucose (after a 16-hour fast, in mmol/L) multiplied by fasting insulin (after a 16-hour fast, in μ U/mL), divided by 22.5.

### Biochemical assays

Plasma biochemical parameters were assessed using commercial ELISA kits, including ALT (mouse ALT ELISA kit, cat. no. ab282882, Abcam), AST (mouse AST ELISA kit, cat. no. ab263882, Abcam), insulin (mouse insulin ELISA kit, cat. no. 10-1247-01, Mercodia), triglycerides (TG assay kit, cat. no. ab65336, Abcam), free fatty acids (FFA assay kit, cat. no. ab65341, Abcam), and corticosterone (CORT ELISA kit, cat. no. ab108821, Abcam). For CORT analysis, plasma was collected at 8:00 AM (during the first hour of the light cycle) under general anesthesia induced by isoflurane inhalation. All assays were performed strictly following the manufacturer’s protocols.

### Mouse ACBP/DBI ELISA

For *in vivo* analyses, plasma samples were collected in lithium heparin tubes (cat. no. 450535, Greiner Bio-One), centrifuged at 1,500 g for 10 minutes, and stored at −80 ${}^{\circ }$ C until use. ACBP/DBI levels were quantified by ELISA. High-binding 96-well plates (Corning) were coated overnight at 4 ${}^{\circ }$ C with 100 μ l/well of murine anti-ACBP/DBI capture antibody (cat. no. ab231910, Abcam) diluted at 1 μ g/ml in PBS. After three washes with 0.1% Tween 20 in TBS, plates were blocked with 100 μ l of blocking buffer (1% BSA, 0.05% Tween 20 in PBS) for 2 hours at room temperature (RT). Plasma samples (typically diluted 1:20, adjusted as needed) were added (100 μ l per well) and incubated for 2 hours at RT. Following three washes, detection was performed using 100 μ l of murine anti-ACBP/DBI antibody (cat. no. MBS2005521, MyBioSource) diluted at 1 μ g/ml in blocking buffer and incubated for 1 hour at RT. After three additional washes, 100 μ l of HRP-conjugated avidin (1:1,000 in blocking buffer) was applied for 30 minutes at RT. Plates were washed four times, and the signal was developed using 100 μ l of 1-Step Ultra TMB-ELISA substrate (cat. no. 34029, Thermo Fisher Scientific) for 10–30 minutes at RT in the dark. The reaction was terminated with 50 μ l of 2N H ${}_{2}$ SO ${}_{4}$ , and absorbance was measured at 450 nm using a FLUOstar OPTIMA microplate reader.

## AUTHORS CONTRIBUTIONS

H.P. and Z.S. performed most of the *in vitro* and *in vivo* experiments. X.D. contributed to *in vivo* experiments. O.K. provided support for preclinical evaluation. G.K. conceived the project and designed the study. G.K. and I.M. supervised the project and wrote the manuscript with input from all other authors.

## CONFLICT OF INTEREST

HP, IM and GK are the inventors of patents covering the therapeutic utility of ACBP/DBI neutralization. OK is a scientific co-founder of Samsara Therapeutics. IM is a consultant for Osasuna Therapeutics. GK has been holding research contracts with Daiichi Sankyo, Eleor, Kaleido, Lytix Pharma, PharmaMar, Osasuna Therapeutics, Samsara Therapeutics, Sanofi, Sutro, Tollys, and Vascage. GK is on the Board of Directors of the Bristol Myers Squibb Foundation France. GK is a scientific co-founder of everImmune, Osasuna Therapeutics, Samsara Therapeutics and Therafast Bio. GK is in the scientific advisory boards of Hevolution, Institut Servier, and Rejuveron Life Sciences/Centenara Labs AG. GK is the inventor of patents covering therapeutic targeting of aging, cancer, cystic fibrosis and metabolic disorders. Among these patents, one “Methods for weight reduction” (US11905330B1) is relevant to this study. GK’s brother, Romano Kroemer, was an employee of Sanofi and now consults for Boehringer-Ingelheim. GK’s wife, Laurence Zitvogel, has held research contracts with Glaxo Smyth Kline, Incyte, Lytix, Kaleido, Innovate Pharma, Daiichi Sankyo, Pilege, Merus, Transgene, 9 m, Tusk and Roche, was on the on the Board of Directors of Transgene, is a cofounder of everImmune, and holds patents covering the treatment of cancer and the therapeutic manipulation of the microbiota. The funders had no role in the design of the study, in the writing of the manuscript, or in the decision to publish the results.

## ABBREVIATIONS

ACBP – acyl-CoA binding protein

CORT – CS induced by oral corticosterone

CS – Cushing syndrome

DBI – diazepam binding inhibitor

GABA – gamma-amino butyrat acid

GABA_A_ – GABA Type A

GCs – glucocorticoids

iBAT – interscapular brown adipose tissue

mABs – monoclonal antibodies

RANKL – receptor activator of NK-κB ligand

WAT – white adipose tissue

GR – GC receptor

## References

[b0069e] Hardy RS, Raza K, Cooper MS (2020). Therapeutic glucocorticoids: Mechanisms of actions in rheumatic diseases. Nat Rev Rheumatol.

[b00711] Chotiyarnwong P, McCloskey EV (2020). Pathogenesis of glucocorticoid-induced osteoporosis and options for treatment. Nat Rev Endocrinol.

[b00777] Li J-X, Cummins CL (2022). Fresh insights into glucocorticoid-induced diabetes mellitus and new therapeutic directions. Nat Rev Endocrinol.

[b007dd] Page-Wilson G, Oak B, Silber A, Okeyo JC, Ortiz N, O’Hara M, Moloney S, Geer EB (2024). Holistic burden of illness in patients with endogenous Cushing’s syndrome: A systematic literature review. Endocrinol Diabetes Metab.

[b0088c] Nieman LK, Castinetti F, Newell-Price J, Valassi E, Drouin J, Takahashi Y, Lacroix A (2025). Cushing syndrome. Nat Rev Dis Primers.

[b00930] Tonon M-C, Vaudry H, Chuquet J, Guillebaud F, Fan J, Masmoudi-Kouki O, Vaudry D, Lanfray D, Morin F, Prevot V, Papadopoulos V, Troadec J-D, Leprince J (2020). Endozepines and their receptors: Structure, functions and pathophysiological significance. Pharmacol Ther.

[b00a1d] Montégut L, Abdellatif M, Motiño O, Madeo F, Martins I, Quesada V, López-Otín C, Kroemer G (2023). Acyl coenzyme A binding protein (ACBP): An aging- and disease-relevant “autophagy checkpoint”. Aging Cell.

[b00acc] Paul M, Nixon M (2024). ACBP orchestrates the metabolic phenotype in Cushing’s syndrome. Nat Metab.

[b00b32] Pan H (2024). Pathogenic role of acyl coenzyme A binding protein (ACBP) in Cushing’s syndrome. Nat Metab.

[b00b8c] Pan H, Tian A-L, Castinetti F, Martins I, Kepp O, Kroemer G (2025). Autophagy-dependent hepatocyte secretion of DBI/ACBP induced by glucocorticoids determines the pathogenesis of Cushing syndrome. Autophagy.

[b00c26] García-Eguren G, Giró O, Romero M del M, Grasa M, Hanzu FA (2019). Chronic hypercortisolism causes more persistent visceral adiposity than HFD-induced obesity. J Endocrinol.

[b00cb3] Bravo-San Pedro JM (2019). Acyl-CoA-binding protein is a lipogenic factor that triggers food intake and obesity. Cell Metab.

[b00d0d] Nogueira-Recalde U, Lambertucci F, Montégut L, Motiño O, Chen H, Lachkar S, Anagnostopoulos G, Stoll G, Li S, Carbonier V, Saavedra Díaz E, Blanco FJ, van Tetering G, de Boer M, Maiuri MC, Caramés B, Martins I, Kroemer G (2025). Neutralization of acyl CoA binding protein (ACBP) for the experimental treatment of osteoarthritis. Cell Death Differ.

[b00e43] Díaz-Catalán D, Capó J, Vega-Beyhart A, Rodrigo-Calvo MT, Mora M, Vidal O, Squarcia M, Enseñat J, Casals G, Hanzu F (2025). Sex-dependent effects of FGF21 on HPA axis regulation and adrenal regeneration after Cushing syndrome in mice. Mol Metabol.

[b00f09] Ma K, Mallidis C, Bhasin S, Mahabadi V, Artaza J, Gonzalez-Cadavid N, Arias J, Salehian B (2003). Glucocorticoid-induced skeletal muscle atrophy is associated with upregulation of myostatin gene expression. Am J Physiol Endocrinol Metab.

[b00fbd] Gilson H, Schakman O, Combaret L, Lause P, Grobet L, Attaix D, Ketelslegers JM, Thissen JP (2007). Myostatin gene deletion prevents glucocorticoid-induced muscle atrophy. Endocrinology.

[b01071] Braun TP, Marks DL (2015). The regulation of muscle mass by endogenous glucocorticoids. Front Physiol.

[b010cf] Piemontese M, Xiong J, Fujiwara Y, Thostenson JD, O’Brien CA (2016). Cortical bone loss caused by glucocorticoid excess requires RANKL production by osteocytes and is associated with reduced OPG expression in mice. Am J Physiol Endocrinol Metab.

[b0115c] Mozo L, Suárez A, Gutiérrez C (2004). Glucocorticoids up-regulate constitutive interleukin-10 production by human monocytes. Clin Exp Allergy.

[b011cf] Gayo A, Mozo L, Suárez A, Tuñon A, Lahoz C, Gutiérrez C (1998). Glucocorticoids increase IL-10 expression in multiple sclerosis patients with acute relapse. J Neuroimmunol.

[b01269] Vago JP, Tavares LP, Riccardi C, Teixeira MM, Sousa LP (2021). Exploiting the pro-resolving actions of glucocorticoid-induced proteins Annexin A1 and GILZ in infectious diseases. Biomed Pharmacother.

[b012ee] Hong E-G, Ko HJ, Cho Y-R, Kim H-J, Ma Z, Yu TY, Friedline RH, Kurt-Jones E, Finberg R, Fischer MA, Granger EL, Norbury CC, Hauschka SD, Philbrick WM, Lee C-G, Elias JA, Kim JK (2009). Interleukin-10 prevents diet-induced insulin resistance by attenuating macrophage and cytokine response in skeletal muscle. Diabetes.

[b01417] Rios-Arce ND, Dagenais A, Feenstra D, Coughlin B, Kang HJ, Mohr S, McCabe LR, Parameswaran N (2020). Loss of interleukin-10 exacerbates early Type-1 diabetes-induced bone loss. J Cell Physiol.

[b014cb] Akasheh RT, Pini M, Pang J, Fantuzzi G (2013). Increased Adiposity in Annexin A1-Deficient Mice. PLOS ONE.

[b01546] Favero V, Cremaschi A, Parazzoli C, Falchetti A, Gaudio A, Gennari L, Scillitani A, Vescini F, Morelli V, Aresta C, Chiodini I (2022). Pathophysiology of mild hypercortisolism: From the bench to the bedside. Int J Mol Sci.

[b0161e] Farah S, Nasr L, Eid Fares J (2024). An overlooked disease: Minimal autonomous cortisol secretion (MACS). A narrative review. Endocr Metab Immune Disord Drug Targets.

[b01691] Szychlińska M, Baranowska-Jurkun A, Matuszewski W, Wołos-Kłosowicz K, Bandurska-Stankiewicz E (2020). Markers of subclinical cardiovascular disease in patients with adrenal incidentaloma. Medicina.

[b0171b] Sagmeister MS, Harper L, Hardy RS (2023). Cortisol excess in chronic kidney disease – A review of changes and impact on mortality. Front Endocrinol.

[b01786] Inoue K, Horikoshi H, Omura M, Tsurutani Y, Saito J, Nishikawa T (2023). Association between aldosterone and hypertension among patients with overt and subclinical hypercortisolism. J Endocr Soc.

[b0181d] Lou Y, Ren L, Chen H, Zhang T, Pan Q (2024). Unveiling the hidden impact: Subclinical hypercortisolism and its subtle influence on bone health. Aging Medicine.

[b018aa] Joseph A (2020). Metabolic and psychiatric effects of acyl coenzyme A binding protein (ACBP)/diazepam binding inhibitor (DBI). Cell Death Dis.

[b01901] Montégut L, Lambertucci F, Moledo-Nodar L, Fiuza-Luces C, Rodríguez-López C, Serra-Rexach JA, Lachkar S, Motiño O, Abdellatif M, Durand S, Aprahamian F, Carbonnier V, Le Corre D, Mouillet-Richard S, Chen H, Sauvat A, Dong Y, Li S, Rong Y, Pietrocola F, Laurent-Puig P, López-Otín C, Martins I, Barcena C, Lucia A, Kroemer G (2025). Acyl-CoA-binding protein as a driver of pathological aging. Proc Natl Acad Sci U S A.

[b01a9a] Montégut L (2024). Acyl-coenzyme a binding protein (ACBP) - A risk factor for cancer diagnosis and an inhibitor of immunosurveillance. Mol Cancer.

[b01af1] Motiño O, Lambertucci F, Joseph A, Durand S, Anagnostopoulos G, Li S, Carbonnier V, Nogueira-Recalde U, Montégut L, Chen H, Aprahamian F, Nirmalathasan N, Maiuri MC, Pietrocola F, Valla D, Laouénan C, Gautier J-F, Castera L, Martins I, Kroemer G (2025). ACBP/DBI neutralization for the experimental treatment of fatty liver disease. Cell Death Differ.

[b01c41] Li S (2025). Neutralization of acyl coenzyme A binding protein for the experimental prevention and treatment of hepatocellular carcinoma. Cell Rep Med.

[b01c96] Motiño O, Lambertucci F, Anagnostopoulos G, Li S, Nah J, Castoldi F, Senovilla L, Montégut L, Chen H, Durand S, Bourgin M, Aprahamian F, Nirmalathasan N, Alvarez-Valadez K, Sauvat A, Carbonnier V, Djavaheri-Mergny M, Pietrocola F, Sadoshima J, Maiuri MC, Martins I, Kroemer G (2022). ACBP/DBI protein neutralization confers autophagy-dependent organ protection through inhibition of cell loss, inflammation, and fibrosis. Proc Natl Acad Sci U S A.

[b01dfb] Montégut L, Joseph A, Chen H, Abdellatif M, Ruckenstuhl C, Motiño O, Lambertucci F, Anagnostopoulos G, Lachkar S, Dichtinger S, Maiuri MC, Goldwasser F, Blanchet B, Fumeron F, Martins I, Madeo F, Kroemer G (2023). High plasma concentrations of acyl-coenzyme A binding protein (ACBP) predispose to cardiovascular disease: Evidence for a phylogenetically conserved proaging function of ACBP. Aging Cell.

[b01f1f] Montégut L, Derosa L, Messaoudene M, Chen H, Lambertucci F, Routy B, Zitvogel L, Martins I, Kroemer G (2024). Benzodiazepines compromise the outcome of cancer immunotherapy. Oncoimmunology.

[b01fdb] Li S, Lambertucci F, Montégut L, Martins I, Pol J, Piacentini M, Maiuri MC, Kroemer G (2025). ACBP/DBI neutralization for the prevention and treatment of malignant and non-malignant liver diseases. Cell Death Dis.

